# Epidemiology, Seasonality and Treatment of Hospitalized Adults and Adolescents with Influenza in Jingzhou, China, 2010-2012

**DOI:** 10.1371/journal.pone.0150713

**Published:** 2016-03-09

**Authors:** Jiandong Zheng, Xixiang Huo, Yang Huai, Lin Xiao, Hui Jiang, John Klena, Carolyn M. Greene, Xuesen Xing, Jigui Huang, Shali Liu, Youxing Peng, Hui Yang, Jun Luo, Zhibin Peng, Linlin Liu, Maoyi Chen, Hui Chen, Yuzhi Zhang, Danqin Huang, Xuhua Guan, Luzhao Feng, Faxian Zhan, Dale J. Hu, Jay K. Varma, Hongjie Yu

**Affiliations:** 1 Division of Infectious Disease, Key Laboratory of Surveillance and Early-warning on Infectious Disease, Chinese Center for Disease Control and Prevention, Beijing, China; 2 Hubei Provincial Centre for Disease Control and Prevention, Wuhan, China; 3 China-US Collaborative Program on Emerging and Re-emerging Infection Disease, Center for Global Health, Centers for Disease Control and Prevention, Beijing, China; 4 Jingzhou Center for Disease Control and Prevention, Jingzhou, China; 5 Global Disease Detection Branch, Division of Global Health Protection, Center for Global Health, Centers for Disease Control and Prevention, Atlanta, United States of America; 6 Influenza Division, U.S. Centers for Disease Control and Prevention, Atlanta, United States of America; 7 Jingzhou Central Hospital, Jingzhou, China; 8 Jingzhou First People’s Hospital, Jingzhou, China; 9 Jingzhou Second People’s Hospital, Jingzhou, China; 10 Jingzhou Maternal and Children's Hospital, Jingzhou, China; 11 Vaccine Research Program, Division of AIDS, NIAID/NIH, Bethesda, United States of America; University Hospital San Giovanni Battista di Torino, ITALY

## Abstract

**Background:**

After the 2009 influenza A (H1N1) pandemic, we conducted hospital-based severe acute respiratory infection (SARI) surveillance in one central Chinese city to assess disease burden attributable to influenza among adults and adolescents.

**Methods:**

We defined an adult SARI case as a hospitalized patient aged ≥ 15 years with temperature ≥38.0°C and at least one of the following: cough, sore throat, tachypnea, difficulty breathing, abnormal breath sounds on auscultation, sputum production, hemoptysis, chest pain, or chest radiograph consistent with pneumonia. For each enrolled SARI case-patient, we completed a standardized case report form, and collected a nasopharyngeal swab within 24 hours of admission. Specimens were tested for influenza viruses by real-time reverse transcription polymerase chain reaction (rRT-PCR). We analyzed data from adult SARI cases in four hospitals in Jingzhou, China from April 2010 to April 2012.

**Results:**

Of 1,790 adult SARI patients enrolled, 40% were aged ≥ 65 years old. The median duration of hospitalization was 9 days. Nearly all were prescribed antibiotics during their hospitalization, less than 1% were prescribed oseltamivir, and 28% were prescribed corticosteroids. Only 0.1% reported receiving influenza vaccination in the past year. Of 1,704 samples tested, 16% were positive for influenza. Influenza activity in all age groups showed winter-spring and summer peaks. Influenza-positive patients had a longer duration from illness onset to hospitalization and a shorter duration from hospital admission to discharge or death compared to influenza negative SARI patients.

**Conclusions:**

There is substantial burden of influenza-associated SARI hospitalizations in Jingzhou, China, especially among older adults. More effective promotion of annual seasonal influenza vaccination and timely oseltamivir treatment among high risk groups may improve influenza prevention and control in China.

## Introduction

According to the World Health Organization (WHO), 5%– 10% of adults and 20%– 30% of children worldwide are infected with the influenza virus each year, resulting in 3 to 5 million cases of severe illness, and 250,000 to 500,000 deaths [1]. China has conducted nationwide influenza-like illness (ILI) surveillance in hospital outpatient and emergency departments since 2000. While ILI surveillance provides useful information on the timing of influenza seasons and the predominating types/sub-types of circulating viruses, comprehensive clinical or epidemiologic data are not collected [2]. After the 2009 influenza A (H1N1) pandemic, WHO stressed the importance of conducting severe acute respiratory infection (SARI) surveillance as a critical component of influenza surveillance to contribute to the understanding of disease burden, and more specifically, to identify groups at high risk for severe disease[3,4].

In November 2009, the Chinese Ministry of Health established a national sentinel SARI surveillance system in ten hospitals within ten cities of China to monitor the clinical severity of the 2009 A(H1N1) pandemic and to understand risk factors for severe illness[5]. Two months later, the Chinese Center for Disease Control and Prevention (China CDC), in collaboration with the United States Centers for Disease Control and Prevention (US CDC), launched a hospital-based SARI surveillance system in Jingzhou city, Hubei Province, to assess hospitalizations attributable to influenza in central China. To date, there are limited published data describing the epidemiology of SARI cases in China.

We analyzed data from the Jingzhou SARI hospital-based surveillance system from April 2010 to April 2012 to characterize the epidemiology and treatment of influenza-confirmed SARI cases among persons aged ≥15 years old in central China.

## Methods

Surveillance was conducted in three general hospitals in Jingzhou City, Hubei Province located in central China as previously described [6]. We collected demographic, clinical, and outcome data for all enrolled hospitalized adult and adolescent SARI patients (aged ≥15 years old) and performed influenza testing for those patients with respiratory specimens to characterize the epidemiology of SARI attributable to influenza between April 5, 2010 and April 8, 2012.

### Case definitions

We defined an adult SARI case as any person ≥15 years old admitted to one of the three surveillance hospitals with acute onset of self-reported fever (≥38.0°C) before hospital admission, or physician-measured fever (rectal or axillary temperature ≥38.0°C) in the outpatient/emergency or inpatient department and at least one of the following: cough, sore throat, tachypnea, difficulty breathing, abnormal breath sounds on auscultation, sputum production, hemoptysis, chest pain, or chest radiograph consistent with pneumonia. We defined a laboratory-confirmed influenza case as a SARI case-patient with a nasopharyngeal swab or a nasopharyngeal aspirate specimen that tested positive for influenza by real-time reverse transcription polymerase chain reaction (rRT-PCR) assay. A severe SARI case was defined as a SARI patient admitted to the intensive care unit (ICU) during hospitalization or who died during hospitalization or within 30 days after discharge, if the death was associated with the recent SARI hospitalization.

### Data Collection

After obtaining verbal consent, physicians enrolled eligible case patients meeting the SARI case definition upon or during hospitalization. Physicians then reviewed case patients’ medical records to abstract demographic information, past medical history, clinical signs and symptoms of current illness, and radiographic data using a structured case report form (CRF). After discharge, physicians were required to update the CRF with information about treatment, disease outcomes and influenza testing results. Local Jingzhou Center for Disease Control and Prevention (Jingzhou CDC) staff followed-up with case-patients by phone 30 days after discharge to assess whether patients had survived at least one month post-discharge.

### Laboratory testing

Nurses collected nasopharyngeal swabs from SARI case-patients within 24 hours of enrollment following standardized procedures [7]. Specimens were transferred to the Jingzhou CDC laboratory where they were tested for influenza A, B, and influenza A virus subtypes [H1, (H1N1)pdm09, H3] by rRT PCR. Handling, processing and testing of the specimens have been described in detail elsewhere [6]. Test results for the rRT PCR were to be returned to the treating physicians within one week of specimen delivery.

### Analysis

Data were entered using EpiData software (Version 3.1), converted into SPSS format, and analyzed using SPSS (v17.0, SPSS, Chicago, IL, USA). Descriptive statistics, including frequency analysis for categorical variables, and medians and interquartile ranges (IQRs) for continuous variables, were calculated. To further identify potential risk factors for severe SARI, we selected variables with p<0.05 on univariate analysis in addition to those we regarded as potential risk factors for severe SARI to include in a multiple logistic regression.

### Human subjects review

Study participants meeting the case definition for SARI were not asked to provide written informed consent for the reason that China’s Ministry of Health (now integrated into “National Health and Family Planning Commission”) issued a national surveillance protocol for SARI in October 2009 in response to the 2009 H1N1 pandemic [5]. For this project as part of the national SARI surveillance system, hospitals and public health officials were authorized to collect nasopharyngeal swabs and individual data from patients hospitalized with acute respiratory infections and participation only required patients or their parent/guardian to provide brief verbal consent. The usage of verbal consent for the participants and the consent procedure used for participants under the age of 18 had been approved by the Ethical Review Committee of China CDC (Beijing, China) and the Institutional Review Board “C” of the US CDC (Atlanta, Georgia). We did not participate in collecting individual data from enrolled SARI patients and had access to the surveillance database for analysis without patients’ name.

## Results

### Characteristics of enrolled adult SARI patients

From April 5, 2010 to April 8, 2012, we enrolled a total of 1,790 adult SARI case patients. Of those, 60% were male, and 40% were aged 65 years and older. The four most common signs and symptoms upon enrollment included cough (77%), abnormal breath sounds on auscultation (57%), temperature ≥38.0°C(56%) and sputum production (49%). Chest x-ray was performed on 90% of case-patients, and, among these, 75% had radiographic evidence of pneumonia. The median duration of hospitalization was 9 days among all case-patients, and increased with age. Median hospitalization stay was 7 days, 9 days and 11 days for those aged 15–49 years, 50–64 years, and ≥ 65 years, respectively. Seven percent of SARI case-patients were admitted to the ICU during their hospitalization, and, among all SARI case-patients, 4% died during hospitalization or within 30 days after discharge. Forty-five percent of SARI case-patients reported taking antibiotics for their illness before hospitalization, while nearly 100% were prescribed antibiotics during their hospitalization, less than 1% were prescribed oseltamivir, and 28% were prescribed corticosteroids. Nearly half (48%) of enrolled case-patients had at least one underlying medical condition, the most common being hypertension (18%), chronic bronchitis (11%), and chronic obstructive pulmonary disease (8%). The proportions of case-patients that reported contact with a person with fever or respiratory symptoms, a sick or dead bird, or a recent visit to a live poultry market were all < = 2%. (Table 1)

**Table 1:** Characteristics of adult severe, acute respiratory infection (SARI) patients and those with laboratory-confirmed influenza virus infection in Jingzhou, China, April 5, 2010 –April 8, 2012.

Characteristics^1^	All adult SARI patients (n = 1,790)	Adult SARI patients with influenza virus infection (n = 281)	Adult SARI patients without influenza virus infection (n = 1,423)	p value
***Male sex***	1,062 (60)	170 (60)	835 (59)	0.571
***Age group***				0.609
15–49 years	617 (35)	93 (33)	495 (35)	
50-64years	454 (25)	77 (27)	360 (25)	
≥65 years	719 (40)	111 (40)	568 (40)	
***underlying medical condition***				
At least one^2^	866 (48)	145 (52)	667 (47)	0.147
hypertension	314 (18)	40 (14)	249 (17)	0.183
Chronic bronchitis	199 (11)	34 (12)	158 (11)	0.629
Chronic obstructive pulmonary disease	148 (8)	27 (10)	111 (8)	0.310
Cardiovascular disease	116 (6)	20 (7)	88 (6)	0.557
Diabetes	86 (5)	12 (4)	71 (5)	0.609
Asthma	61 (3)	17 (6)	42 (3)	*0*.*009*
Obesity^3^	16/1,737 (1)	3/278 (1)	12/1,378 (1)	0.947
***Received seasonal trivalent influenza vaccination in the past year***				*0*.*100*
Yes	2 (0)	1 (0)	1 (0)	
No	1,053 (59)	150 (54)	841 (59)	
Unknown	735 (41)	130 (46)	581 (41)	
***Exposure history***				
Currently smoke	259 (14)	42 (15)	204 (14)	0.*790*
Contact with anyone else with fever or respiratory symptoms in the past two weeks^4^				0.536
Yes	21 (1)	4 (1)	15 (1)	
No	967 (54)	142 (51)	767 (54)	
Unknown	802 (45)	135 (48)	641 (45)	
Contact with sick or dead poultry in the past two weeks				0.375
Yes	1 (0)	0 (0)	1 (0)	
No	1,079 (60)	157 (56)	855 (60)	
Unknown	710 (40)	124 (44)	567 (40)	
Visit live poultry market in the past two weeks				0.518
Yes	38 (2)	6 (2)	25 (2)	
No	1,013 (57)	149 (53)	806 (57)	
Unknown	739 (41)	126 (45)	592 (42)	
***Symptoms at hospital admission***				
Cough	1,382 (77)	249 (89)	1,062 (75)	*<0*.*001*
Sputum production	874 (49)	171 (61)	653 (46)	*<0*.*001*
Sore throat	340 (19)	58 (21)	259 (18)	0.337
Dyspnoea	250 (14)	54 (19)	184 (13)	*0*.*005*
Rhinorrhoea	86 (5)	18 (6)	62 (4)	0.138
***Signs at hospital admission***				
Temperature ≥38.0°C	997 (56)	167 (59)	797 (56)	0.290
Abnormal breath sounds on auscultation	1,023 (57)	178 (63)	806 (57)	*0*.*038*
Shortness of breath	225 (13)	35 (13)	181 (13)	0.903
***Had chest radiograph performed***	1,619 (90)	257 (92)	1,282 (90)	0.479
Radiographic diagnosis of pneumonia	1,216/1,619 (75)	183/257 (71)	979/1,282 (76)	0.079
***Treatment***				
Reported taking antibiotics for current illness prior to hospital admission				0.106
Yes	806 (45)	136 (48)	622 (44)	
No	587 (33)	77 (27)	482 (34)	
Unknown	397 (22)	68 (24)	319 (22)	
Received antibiotics during hospitalization	1782 (100)	281 (100)	1415 (99)	0.208
Received corticosteroid treatment during hospitalization	503 (28)	88 (31)	389 (27)	0.174
Received oseltamivir during hospitalization	5 (0)	1 (0)	3 (0)	1.000
Admitted to ICU^5^	133 (7)	21 (8)	108 (8)	0.946
***Clinical course*, *median days (IQR***^6^***)***				
From illness onset to hospital admission	1 (4)	2 (4)	1 (4)	*0*.*030*
From hospital admission to discharge or death	9 (8)	8 (7)	9 (8)	*0*.*036*
***Clinical outcome***				
Died during hospitalization	26 (2)	3 (1)	20 (1)	0.654
Died within 30 days after discharge	28 (2)	4 (1)	24 (2)	0.751

^1^Data is presented as no. (%) of patients unless otherwise indicated. Denominators for testing of fewer patients than full group are indicated. Percentages may not total 100 because of rounding.

^2^ At least one underlying medical condition defined as an admission diagnosis of any of the following: hypertension, chronic bronchitis, chronic obstructive pulmonary disease, cardiovascular disease, diabetes, asthma, Tuberculosis, or renal dysfunction.

^3^Body-mass index (BMI) was calculated for patients with available height and weight data to assess obesity (defined as BMI>28)

^4^Contact with anyone else with fever or respiratory symptoms defined as having been in close contact (within one meter) or direct contact with a person with fever or respiratory symptoms

^5^ICU, intensive care unit.

^6^IQR, interquartile range.

### Characteristics of adult SARI patients with laboratory confirmed influenza

Of 1,704 samples from SARI case patients, 16% were positive for influenza. The proportion of influenza positive cases among SARI cases was similar among men and women (17%, 170/1,005 *vs*. 16%, 111/699; p = 0.321), and among different age groups. Adults aged ≥65 years accounted for the largest proportion of influenza-positive SARI cases (40%), followed by those aged 15–49 years (33%) and 50–64 years (27%). Adult influenza-positive SARI patients most often presented with cough (89%), abnormal breath sounds on auscultation (63%), sputum production (61%), and temperature ≥38.0°C at admission (59%). Compared with influenza-negative patients, influenza-positive patients more often presented with cough (89% vs 75%, p<0.001), abnormal breath sounds on auscultation (63% vs 57%, P = 0.038), sputum production (61% vs 46%, p<0.001) and dyspnea (19% vs 13%, P = 0.005). Influenza-positive patients also had a longer duration from illness onset to hospitalization (2 [IQR = 4] days vs. 1 [IQR = 4] day, p = 0.030) and a shorter duration from hospital admission to discharge/death (8 [IQR = 7] days vs. 9 [IQR = 8] days, p = 0.036). A similar proportion of influenza-positive and influenza-negative patients had at least one underlying chronic medical condition (52% *vs*. 47%, p = 0.147), and while the most common underlying conditions were similar in both groups, a greater proportion of influenza-positive patients had asthma (6% *vs*. 3%, p = 0.009). Known influenza vaccination within the prior year in both groups was close to 0%, and, in both groups, more than 40% did not know their influenza vaccination status. (Table 1)

### Influenza activity among adult SARI cases

**Fig 1:**
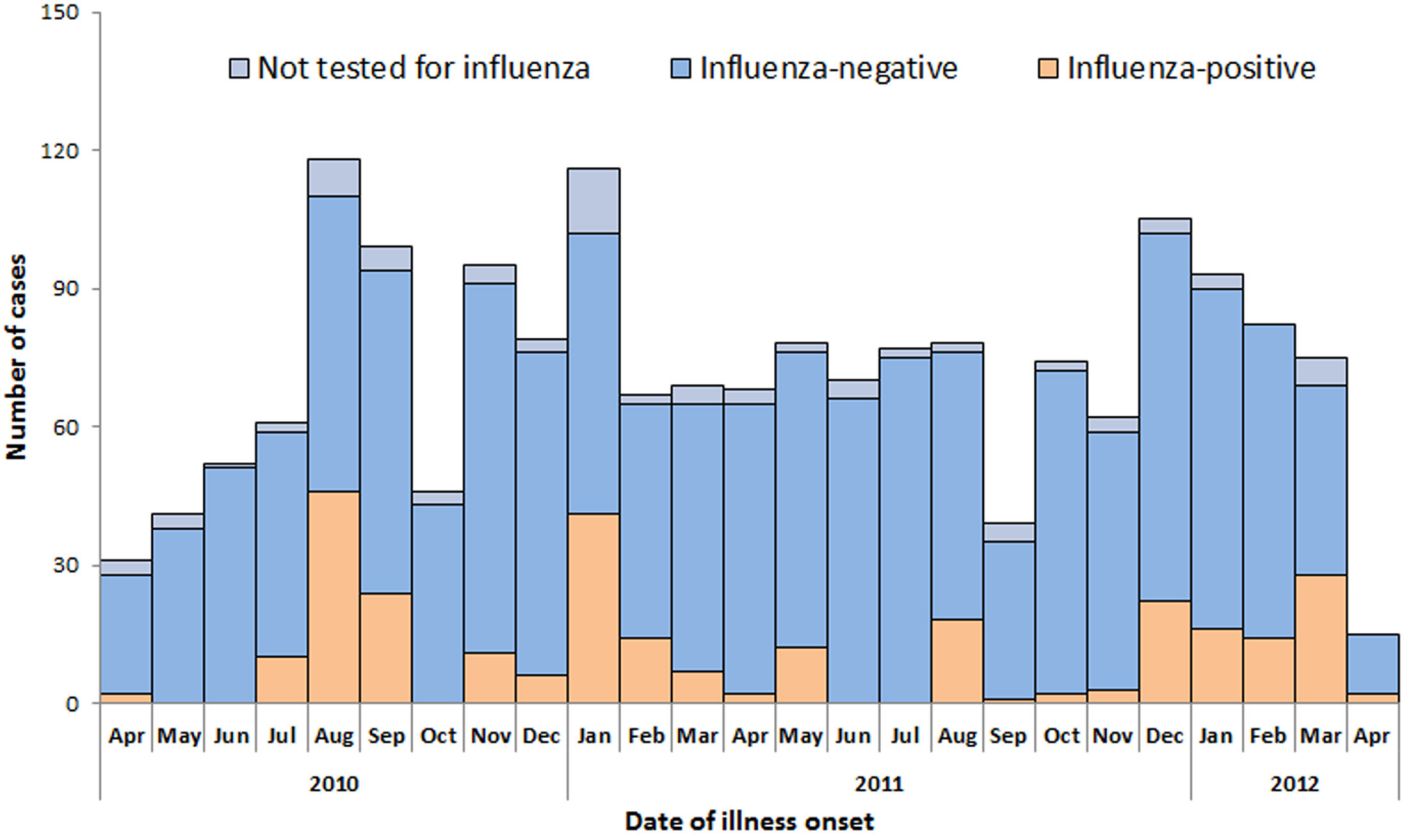
Influenza test results among adults (aged ≥15 years) hospitalized with severe acute respiratory infection (N = 1,790) by date of illness onset, Jingzhou, China, April 5, 2010 to April 8, 2012.

**Fig 2:**
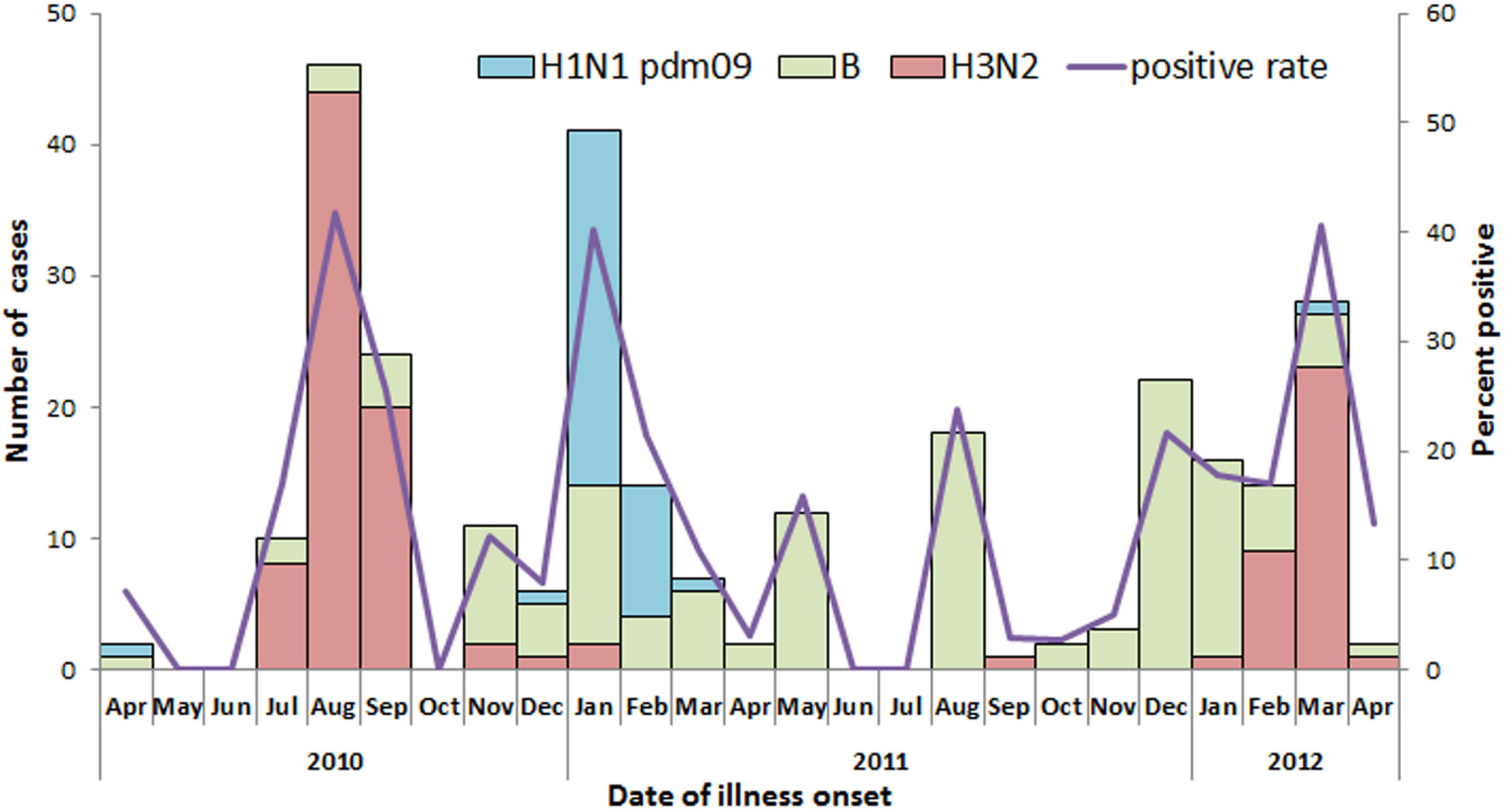
Number and percent of influenza positive by type/subtype and by month of illness onset among adults (aged ≥15 years) hospitalized with severe acute respiratory infection (n = 1,790), Jingzhou, China, April 5, 2010 to April 8, 2012.

The number of enrolled SARI cases during the 24-month surveillance period peaked during August-September and January in 2010–2011 and during December-February in 2011–2012 (Fig 1). Influenza activity in all age groups showed winter-spring and summer peaks during this surveillance period. While A(H3N2) was the predominant influenza virus in the 2010 summer months, a mix of H1N1 pdm09 and B predominated in the 2010–11 winter-spring months, B in the 2011 summer months, and a mix of B and A(H3N2) in the 2011–12 winter-spring months (Fig 2). The predominant influenza virus in each season was consistent across all age groups.

### Predictors for severe adult SARI cases

Of the 1790 enrolled adult and adolescent SARI case-patients, 169 (9%) met the case definition for severe SARI. In univariate analysis, factors significantly associated with severe SARI included age ≥65 years (p<0.001), having at least one underlying medical condition (p<0.001), abnormal breath sounds on auscultation at hospital admission (p = 0.012), shortness of breath at hospital admission (p<0.001), and self-report of not taking antibiotics before hospital admission (p<0.001). A similar proportion of influenza-positive and influenza-negative SARI case-patients had severe SARI (9% vs 10%, p = 0.703). (Table 2)

**Table 2:** Predictors for intensive care unit admission or death among adult patients with severe, acute respiratory infection (SARI) in Jingzhou, China, April 5, 2010 –April 8, 2012.

Characteristics^1^	N	Adult SARI with ICU^2^ admission or death outcome n (%)	p value	Crude OR (95% CI)	Adjusted OR (95% CI)
***Sex***					
Male	1,062	112 (11)	0.054	1.388 (0.994, 1.938)	-
Female	728	57 (8)			
***Age***					
≥65 years	719	105 (15)	<0.001	2.691 (1.941, 3.730)	2.128 (1.416, 3.197)
<65years	1,071	64 (6)			
***At least one underlying medical condition***^3^					
Yes	866	110 (13)	<0.001	2.133 (1.532, 2.970)	-
No	924	59 (6)			
***Obesity***^4^					
Yes	16	0 (0)	0.261	-	-
No	1,721	126 (7)			
***Currently smoke***					
Yes	259	24 (9)	0.917	0.976 (0.620, 1.536)	-
No	1,531	145 (9)			
***Temperature*** ***≥38*.*0°C at hospital admission***					
Yes	997	91 (9)	0.610	0.921 (0.670, 1.265)	-
No	793	78 (10)			
***Abnormal breath sounds on auscultation at hospital admission***					
Yes	1,023	112 (11)	0.012	1.531 (1.097, 2.138)	-
No	767	57 (7)			
***Shortness of breath at hospital admission***					
Yes	225	41 (18)	<0.001	2.502 (1.704, 3.672)	1.971 (1.218, 3.189)
No	1,565	128 (8)			
***Reported receiving antibiotics before hospital admission***^†^					
Yes	806	52 (6)	<0.001	0.501 (0.345, 0.729)	0.632 (0.423, 0.945)
No	587	71 (12)			
***Received oseltamivir during hospitalization***^†^					
Yes	5	0 (0)	0.476	-	-
No	1,702	157 (9)			
***Received seasonal trivalent influenza vaccination in the past year***^†^					
Yes	2	0 (0)	0.673	-	-
No	1,053	86 (8)			
***Influenza virus infection***^‡^					
Yes	281	25 (9)	0.703	0.917 (0.586, 1.433)	-
No	1,423	137 (10)			

^1^Data is presented as no. (%) of patients unless otherwise indicated. Denominators for testing of fewer patients than full group are indicated. Percentages may not total 100 because of rounding.

^2^ICU, intensive care unit.

^3^ At least one underlying medical condition defined as: an admission diagnosis of any of the following: hypertension, chronic bronchitis, chronic obstructive pulmonary disease, cardiovascular disease, diabetes, asthma, Tuberculosis, or renal dysfunction.

^4^Body-mass index (BMI) was calculated for patients with available height and weight data to assess obesity (defined as BMI>28)

^†^Missing data and “unknown” responses were excluded

^‡^Cases not tested for influenza virus were excluded.

In the multiple logistic regression model, two risk factors were significantly associated with severe SARI: age ≥65 years (adjusted OR [AOR] = 2.128, 95% CI: 1.416 to 3.197, p<0.001) and shortness of breath at hospital admission (AOR = 1.971, 95% CI: 1.218 to 3.189,p = 0.005). Those who self-reported taking antibiotics before hospital admission were significantly less likely to have severe SARI (AOR = 0.632, 95% CI: 0.423 to 0.945, p = 0.024). (Table 2)

## Discussion

We describe influenza-confirmed SARI cases among persons aged ≥ 15years from the first hospital-based SARI surveillance system in central China. While there were differences in illness presentation and duration of hospitalization among adult SARI case-patients with and without influenza, both groups had similar proportions of severe SARI, were unlikely to receive antiviral treatment, and were equally unlikely to have been vaccinated with seasonal influenza vaccine in the prior year. The proportion receiving antibiotics and corticosteroids during their hospitalization among both influenza positive and negative case-patients was high. Though there was influenza activity year-round, there were two distinct influenza peaks, in winter-spring and summer.

Although 40% of influenza positive SARI cases were among adults aged ≥65 years, there was no significant difference in the proportion of influenza-positive cases between age groups. The four most common signs and symptoms at presentation were the same for SARI patients with and without influenza, and included cough, abnormal breath sounds on auscultation, temperature ≥38.0°C and sputum production. Compared with influenza-negative patients, influenza-positive SARI cases had a longer duration from illness onset to hospitalization and a shorter duration from hospitalization to discharge/death. This difference might reflect a less acute presentation or less severe complications associated with respiratory illness caused by influenza virus infection compared with other etiologic agents of SARI. For example, respiratory syncytial virus (RSV), one of the most common respiratory pathogens causing influenza-like illness and pneumonia [8,9],was estimated to cause 60–80% more deaths in the community than influenza in one regression modeling analysis conducted in England and Wales [10].

Our data reveal that a history of asthma was more common among adult SARI case-patients with confirmed influenza, suggesting that adults with asthma may be at greater risk for SARI if they have influenza compared to other etiologies of SARI. Although previous studies identified asthma as a risk factor in the general population during the 2009 A(H1N1) pandemic [11,12], asthma was not more commonly associated with SARI due to influenza vs SARI due to other etiologies. Data from China’s sentinel SARI surveillance system showed that the prevalence of asthma was lower among SARI case-patients with confirmed influenza compared to those without influenza [13]. Additional studies are needed to better understand asthma as a potential risk factor for influenza-associated SARI hospitalization.

China CDC has recommended annual seasonal influenza vaccination for groups at risk of severe illness from influenza, including pregnant women, young children, the elderly, persons with chronic medical conditions and healthcare personnel [14]. In addition, influenza vaccine effectiveness against medically-attended influenza and influenza-associated hospitalization has been shown to be moderate to high in both the north and south of China [15,16]. However, seasonal influenza vaccination is not included in China’s national immunization program; in most regions of the country, healthcare workers do not routinely recommend seasonal influenza vaccination, and individuals who receive the vaccine must pay for it out-of-pocket. A prior retrospective study showed that less than 2% of the Chinese population was vaccinated for seasonal influenza during the 2008–2009 influenza season [17]. In our study, even though 59% of the patients should have been vaccinated based on either having at least one underlying condition (48%) or being aged > = 65 years (40%), only 2 of the 1790 (0.1%) adult SARI case-patients reported known influenza vaccination within the past year. Further, more than 40% did not know their influenza vaccination status. These data suggest a need for health education on the benefits of seasonal influenza vaccination among adults in central China at high risk for severe illness from influenza or influenza-related complications, and perhaps more importantly, among their health care providers who play a critical role in vaccine uptake [18,19].

The multiple logistic regression model indicated that age ≥65 years was a risk factor associated with severe SARI. Prior studies have demonstrated that people aged ≥65 years old and with at least one underlying medical condition were at high risk of *severe* influenza-related complications [20].The finding of apparent lower risk of developing severe SARI associated with reported antibiotic use prior to hospital admission is interesting and concerning. One potential explanation is that a proportion of SARI cases caused by bacterial pathogens, such as *Streptococcus pneumoniae* or *Haemophilus influenzae* type B (Hib), were sensitive to the antibiotics taken, and thus patients had initiated partially effective treatment before hospitalization. However, our data showed that even among the 281 influenza-infected SARI patients, those who reported taking antibiotics before hospitalization were also less likely to develop severe SARI than those who did not (3%, 4/136 vs. 16%, 12/77; p = 0.001). The issue of appropriate antibiotic use deserves further investigation. In our study, the antibiotic use prior to hospitalization was self-reported, and thus ascertainment of antibiotic use may have been biased. Further, it is unclear what proportions of antibiotic use were appropriate versus inappropriate.

Antiviral treatment within 2 days of influenza illness onset reduces the probability of influenza-related serious complications, including mortality in adults, and the duration of influenza virus shedding [21–24]. A meta-analysis showed that neuraminidase inhibitor treatment of hospitalized adults with influenza A(H1N1)pdm09 virus infection reduced mortality, even when treatment was started after 2 days of illness onset [24]. A retrospective study of hospitalized adults with seasonal influenza in Hong Kong, Beijing, and Singapore reported that neuraminidase inhibitor treatment was independently associated with reduced mortality [25]. The US CDC advises that all patients with suspected or confirmed influenza who are hospitalized, present with severe illness, or are at higher risk for influenza-associated complications regardless of illness severity should receive early antiviral treatment [26]. In our study, while 1,114 (62%) of the 1790 SARI patients were hospitalized within 2 days of illness and thus would have potentially benefitted from early antiviral treatment, only 5 received oseltamivir treatment during their hospitalization. In China, oseltamivir is not prescribed routinely and is usually only administered when hospitalized patients with confirmed influenza are in critical condition. This practice delays treatment to well beyond the time when it would decrease illness severity. Of the 281 SARI case-patients with confirmed influenza in our study, only one was prescribed oseltamivir during hospitalization. Although we do not have data on when the treating physicians received the rRT PCR results for the case-patients, the standard operating procedure required reporting test results back to the treating physician within one week of specimen delivery. As the median number of days from hospital admission to discharge or death for laboratory-confirmed influenza cases was 8 with an interquartile range of 7 days, it is likely that many treating physicians received the laboratory confirmation of influenza during their patient’s hospitalization, but still chose not to prescribe oseltamivir. In contrast, 100% of enrolled SARI case-patients with influenza received empiric antibiotic treatment during their hospitalization, presumably to prevent secondary bacterial infection even if the clinicians knew that the patients were primarily infected with non-bacteria pathogen. The universal use of antibiotics among hospitalized patients with influenza is concerning, given that increasing antimicrobial resistance in both hospital and community settings is an international public health concern [27].

Thirty percent of all SARI patients received corticosteroid treatment during their hospitalization. The use of corticosteroids in the treatment of respiratory infections is controversial. While corticosteroid use has been shown in some studies to reduce mortality among patients with very severe respiratory illness [28–30], and to reduce treatment failure among patients with severe community-acquired pneumonia (CAP) and a high initial inflammatory response [31], a meta-analysis showed no treatment benefit from corticosteroids among all patients with CAP [32]. Early corticosteroid treatment of influenza A(H1N1)pdm09 virus infection was associated with critical illness and death in China [33]. Corticosteroid treatment of hospitalized adults with seasonal influenza in Hong Kong, Beijing, and Singapore was associated with an increased risk of bacterial superinfection and mortality [25]. A meta-analysis reported that corticosteroid treatment of hospitalized patients with influenza A(H1N1)pdm09 virus infection was associated with increased mortality [34]. Furthermore, the use of high-dose corticosteroids can lead to irreversible adverse effects, such as osteonecrosis of the femoral head (ONFH). A retrospective study of SARS survivors in China during 2003 showed that the incidence of ONFH increased from 12.5% among patients receiving one type of corticosteroid during treatment, to 28.6% and 37.1% among patients receiving two and three types of corticosteroids respectively [35]. Although our study did not collect extensive clinical data on SARI patients, our findings show that while 30% received corticosteroid treatment for SARI, only 7% of patients were admitted to the ICU during their hospitalization. Corticosteroids such as low-dose hydrocortisone are only recommended for critically ill patients with suspected adrenal insufficiency or refractory septic shock [36,37]; since it is unlikely that a substantial proportion of our patients had these complications, corticosteroid treatment of both influenza positive and negative SARI patients was probably overused. Further studies, guidelines, and education of physicians about the harms of corticosteroid use in SARI and influenza patients are needed in China.

The Chinese surveillance network for ILI conducted in hospital outpatient and emergency departments has shown that in southern China, seasonal influenza is prevalent throughout the year with a dominant peak in the summer and a less pronounced peak in the winter[38]. This study demonstrated the same seasonality of influenza activity among all age-groups ≥ 15 years old of SARI inpatients in Jingzhou (data not shown). There was increased influenza activity during the cold months of December-January and during the hot summer month of August. There were very few cases of influenza SARI cases due to H1N1 pdm09 in the 2010 calendar year. Influenza B virus was common in both studied influenza seasons, and predominant in the 2011–12 season, indicating that B virus accounted for a major proportion of influenza disease burden among SARI patients during the study period [39].

Our findings are subject to several limitations. First, the case definition for SARI used in this study may not have captured all patients with severe respiratory disease nor those with influenza who presented with non-respiratory symptoms (ie, congestive heart failure, cerebral vascular accident). Second, we relied upon clinicians (i.e., doctors and nurses) within the hospitals to enroll all SARI case-patients. It is likely that some patients meeting our SARI case definition were not captured due to clinician fatigue or lack of time. Third, specimens were not collected from all the enrolled adult SARI case-patients and thus the findings we describe may not be fully representative of all hospitalized SARI cases in Jingzhou during the surveillance period. In addition, we did not collect data on when clinicians received laboratory results confirming influenza in their hospitalized patients. It is likely that longer laboratory turnaround times contributed to clinicians’ limited use of oseltamivir. Finally, in this study we did not test for other viral and bacterial etiologies of SARI.

## Conclusions

Our results highlight the substantial burden of influenza-associated SARI among adolescents and adults in China, in addition to the vast underutilization of seasonal influenza vaccination and the near absence of anti-viral treatment for those with influenza-associated SARI. More effective promotion of annual seasonal influenza vaccination and timely oseltamivir treatment among high risk groups may improve influenza prevention and control in China. Further, our study suggests a need for further study of existing practices of empiric antibiotic treatment and corticosteroid treatment in the absence of evidence-based clinical indications.
